# Control of the Nucleation Density of Molybdenum Disulfide in Large-Scale Synthesis Using Chemical Vapor Deposition

**DOI:** 10.3390/ma11060870

**Published:** 2018-05-23

**Authors:** Haitao Xu, Weipeng Zhou, Xiaowu Zheng, Jiayao Huang, Xiliang Feng, Li Ye, Guanjin Xu, Fang Lin

**Affiliations:** 1 College of Electronic Engineering, South China Agricultural University, Guangzhou 510642, China; xuhaitao@scau.edu.cn (H.X.); weipengzhou@stu.scau.edu.cn (W.Z.); xiaowuzheng@stu.scau.edu.cn (X.Z.); huangjiayao@casachina.com.cn (J.H.); fengxiliang@stu.scau.edu.cn (X.F.); yel17@mails.tsinghua.edu.cn (L.Y.); xuguanjin@stu.scau.edu.cn (G.X.); 2 State Key Laboratory of Silicon Materials, School of Materials Science and Engineering, Zhejiang University, Hangzhou 310027, China

**Keywords:** 2D MoS_2_ crystal, chemical vapor deposition, nucleation density, carrier gas flow rate

## Abstract

Atmospheric pressure chemical vapor deposition (CVD) is presently a promising approach for preparing two-dimensional (2D) MoS_2_ crystals at high temperatures on SiO_2_/Si substrates. In this work, we propose an improved CVD method without hydrogen, which can increase formula flexibility by controlling the heating temperature of MoO_3_ powder and sulfur powder. The results show that the size and coverage of MoS_2_ domains vary largely, from discrete triangles to continuous film, on substrate. We find that the formation of MoS_2_ domains is dependent on the nucleation density of MoS_2_. Laminar flow theory is employed to elucidate the cause of the different shapes of MoS_2_ domains. The distribution of carrier gas speeds at the substrate surface leads to a change of nucleation density and a variation of domain morphology. Thus, nucleation density and domain morphology can be actively controlled by adjusting the carrier gas flow rate in the experimental system. These results are of significance for understanding the growth regulation of 2D MoS_2_ crystals.

## 1. Introduction

As one kind of transition metal dichalcogenide (TMD) [1], molybdenum disulfide (MoS_2_) is the best known material for two-dimensional (2D) crystal research after graphene [2]. With its reduced number of layers, MoS_2_ exhibits many excellent properties [3,4], such as good optical transparency [5], high electron mobility (up to 200 cm^2^/V·s), and direct band-gap structure (Eg = 1.8 eV) [6]. It can be employed to fabricate field effect transistors (FETs) with a high current on/off ratio [5,7], sensitive photodetectors [8,9], light emitting diodes (LEDs) [10,11], and heterojunction solar cells [12,13]. Thus, due to its unique semiconductor properties and wide applications, 2D MoS_2_ attracts great attention. It is considered a potential candidate in atomic-scale semiconductor science [14,15].

Recently, the main preparation methods of MoS_2_ have included hydrothermal synthesis [16], tape auxiliary mechanical exfoliation [17], liquid-phase exfoliation [14], physical vapor deposition (PVD) [18], and chemical vapor deposition (CVD) [19]. Compared with other methods, CVD is an efficient method to massively synthesize an MoS_2_ coating. The CVD method can also alter the shape of MoS_2_ domains from triangular nanosheets to continuous films by controlling synthesis parameters, such as the quantity of the reactants, the temperature of precursors, and the carrier gas flow rate. Previous studies have proven that the nucleation density of MoS_2_ played a key role in the deposition process, leading to the quality and shape control of MoS_2_ domains [20,21,22,23,24,25]. It is reported that, before the growth of MoS_2_, adding a “seed layer” on the substrates can adjust the nucleation density of MoS_2_ and control the shape of MoS_2_ domains [20,21,22]. With its hexagonal lattice structure, graphene can be chosen as a seed layer to form MoS_2_ nuclei. However, the pre-treatment process of adding a seed layer requires the addition of steps to the CVD method, and part of the seed material is toxic [21]. The rest of the seed compound will introduce an unfavorable factor in changing the electronic or optical characteristics of MoS_2_ samples. Hence, it is necessary to find a simplified process to control the nucleation density of MoS_2_.

In this paper, we propose a simple CVD method at atmospheric pressure without hydrogen which can increase formula flexibility by controlling the heating temperature of MoO_3_ powder and sulfur powder. The results show that the size of MoS_2_ grown domains is 10 μm with fast growth. The shape of MoS_2_ domains vary widely, from discrete darts and triangles to continuous film, on substrate. We deduce that the carrier flow rate distribution in the quartz tube leads to a variation in the nucleation density of MoS_2_, resulting in the eventual shape distribution of MoS_2_ domains. Therefore, controlling the carrier gas flow rate can be an effective approach to controlling the shape and coverage of MoS_2_ domains in the CVD method. These results are of significance for understanding the growth regulation of 2D MoS_2_.

## 2. Materials and Methods

### 2.1. Synthesis Precursor

**Figure 1:**
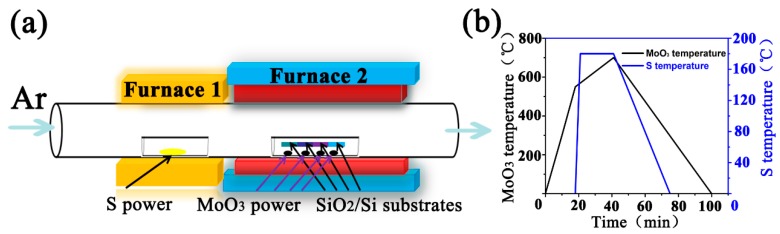
(**a**) Schematic of the CVD (chemical vapor deposition) experimental device. (**b**)Temperature control process of MoO_3_ and S in the CVD system.

As shown in Figure 1, high-purity sulfur (S) and molybdenum trioxide (MoO_3_) powder (Alfa Aesar, >99.9%) were used as raw materials for the synthesis of MoS_2_. SiO_2_/Si substrates were ultra-sonically cleaned with alcohol and deionized water. MoO_3_ powder (0.1 mg) was grounded into four average parts by SiO_2_/Si substrates and placed in a double-open quartz boat a small distance apart from another. Another four pieces of clean SiO_2_/Si substrates were placed on the top of quartz boat, which were seated face-down to the MoO_3_ powder. All of them were situated in the center position of furnace 2. Then, S powder (10 mg) was placed in another double-open quartz boat. They were also transferred into the center of furnace 1. To ensure the vapor concentration of S can be distributed equally on each slice of SiO_2_/Si substrate, we set the distance between the two quartz boats at 24 cm. We employed high-purity (99.999%) argon (Ar) as a carrier gas to avoid the oxidation of MoS_2_ products and control the reaction rates during synthesis. The gas flow rate was precisely controlled by a commercial gas flow controller.

### 2.2. Synthesis Procedure

Atmospheric pressure CVD method is used to prepare MoS_2_ samples. The schematic of the CVD system configuration was shown in Figure 1a. The programming of the temperature control process of furnace was shown in Figure 1b.

The synthesis procedure included two steps. In step 1, while keeping a flow rate of 100 sccm, MoO_3_ was heated to 550 °C at a constant rate of temperature (29 °C/min) in an Ar atmosphere. In step 2, to carefully control evaporation, MoO_3_ was slowly heated to 700 °C (6.8 °C/min) and the temperature kept at 700 °C for 1 min. After step 1, S power was heated rapidly to 180 °C in furnace 1. After 23 min of sulfurization, the furnaces were shut down and the samples were cooled down to room temperature.

### 2.3. Characterizations

Optical microscope (OM) images of MoS_2_ domains were observed using the Nikon Eclipse Ti-U (Nikon, Tokyo, Japan) and Mshot MJ30 (Mshot, Guangzhou, China). Scanning electron microscopy (SEM) images were acquired using a FEI Quanta 450. Raman (FEI Quanta, Hillsboro, OR, USA) and photoluminescence (PL) spectroscopy were performed using a Renishaw inVia Reflex system (Renishaw, Wharton Andech, UK) with a Leica dark-field microscope. The wavelength of the excitation laser was 532 nm, and the focus diameter was approximately 1 μm. The surface feature and film thickness of MoS_2_ domains were measured by an atomic force microscope (AFM, NTEGRA Spectra, NT-MDT, Moscow, Russia).

## 3. Results and Discussion

**Figure 2:**
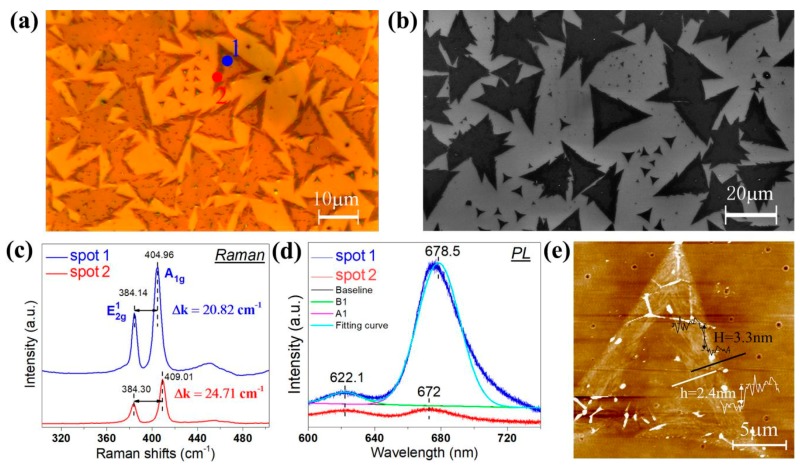
(**a**) Optical images for the MoS_2_ sample. (**b**) SEM (Scanning electron microscopy) images of the MoS_2_ sample. (**c**) The Raman spectroscopy images of the colored circular points corresponding to the areas marked 1 and 2 in (**a**). The laser wavelength was 532 nm. (**d**) The photoluminescence spectroscopy image of the colored circular points corresponding to the two areas marked 1 and 2 in (**a**). The laser wavelength was 532 nm. (**e**) An AFM (atomic force microscope) image of the triangle MoS_2_ sample. The height between the internal position and the edge position of the product (white measurement line, marked h) is 2.4 nm, and the height between the edge position and the SiO_2_/Si substrate (black measuring line, marked H) is 3.3 nm.

The crystal features of MoS_2_ grown on the SiO_2_/Si substrates were analyzed. As shown in Figure 2a, MoS_2_ nanosheets are successfully deposited on the SiO_2_/Si substrate. According to the optical contrast of MoS_2_ nanosheets [23], the film thickness in the inner position of the sample is relatively thinner than that in the edge position. In order to further investigate the surface morphology of the sample, we used SEM to examine the MoS_2_ nanosheets. In Figure 2b, we can find a distinct layered effect where the color depth in the edge position of the sample is deeper than that in the internal position. This is similar to the thickness distribution of MoS_2_ nanosheets observed using OM.

To further confirm the number of layers, we chose two spots in the sample to be characterized by Raman and PL spectroscopy. One spot (blue spot) is in the internal position (region 1) and the other spot (red point) is in the edge position (region 2). It was found that there were two obvious Raman peaks in Figure 2c. E^1^_2g_ represents the in-plane vibrational mode between the molybdenum atom and the sulfur atom. A^1^_g_ stands for the out-of-plane vibrational mode between sulfur atoms [24]. Δk, the Raman frequency difference between E^1^_2g_ and A^1^_g_, can determine the number of layers [25]. The two Raman peaks of the blue spot are located at 384.14 and 404.96 cm^−1^, so the Δk is 20.82 cm^−1^. This Δk corresponds to monolayer MoS_2_ [26]. Similarly, in the red spot, the two Raman peaks are located at 384.30 and 409.01 cm^−1^, and the Δk is 24.71 cm^−1^, which corresponds to few-layer MoS_2_ [15,26]. With the decreased number of layers, the band-gap of MoS_2_ gradually shifts from the indirect band-gap to the direct band-gap. In terms of Figure 2d, the PL spectra of monolayer MoS_2_ in the blue spot, we can see two resonant points at 678.5 nm (1.82 eV) and 622 nm (1.99 eV). The two resonant points correspond to A_1_ (the maximal splitting valence band) and B_1_ (the minimum conduction band), the direct exciton transition of monolayer MoS_2_. The PL spectra was fitted with Gaussian curves. The full width at half maximum (FWHM) of peak at 678.5 nm is 30.2 nm and that of 622 nm is 23.8 nm. On the other hand, the PL spectra of few-layer MoS_2_ in the red spot show weak PL intensity. The A_1_ peak is at 672 nm (1.84 eV), and the B_1_ peak is at 622 nm (1.99 eV). Furthermore, we used AFM to measure the thickness of the sample. According to the measurement results shown in Figure 2e, the height (marked with a white line) between the internal position and the edge position of the sample is h = 2.4 nm. The height (labeled with a white line) between the edge position and the substrate is H = 3.3 nm. Thus, the height between the internal position and the substrate is about 0.9 nm, which is consistent with the thickness of monolayer MoS_2_ [27].

**Figure 3:**
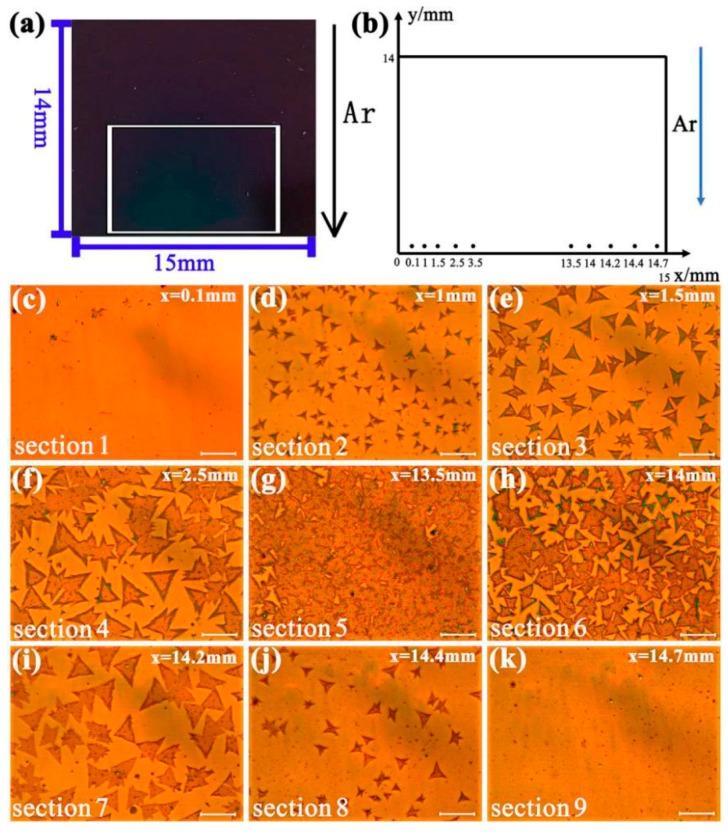
(**a**) Image of the deposition of MoS_2_ on the SiO_2_/Si substrates. (**b**) The Cartesian coordinate map of the selected nine MoS_2_ sample growth regions (**c**–**k**) in (**a**). (**c**–**k**) The optical image of MoS_2_ samples in different growth areas. Scale bar: 20 μm.

The size and coverage of the MoS_2_ domains are highly dependent on the distribution of the samples in the spatial location of the substrate [27]. To better observe this phenomenon, we created an XY-coordinate system where the bottom left corner of a substrate (Figure 3a) is taken as the origin O. As shown in Figure 3b, the y-axis is along the airflow and the x-axis is vertical the airflow. In the rectangular coordinate system, at y = 1.0 mm, 10 points were selected on the x-axis (in the direction of vertical airflow) for observation. According to the distribution characteristics of MoS_2_ domains, nine representative images were selected for display, as shown in Figure 3c–k. At x = 0.1 mm (Figure 3c), owing to the low evaporation concentration of MoO_3_, only small black nuclei appeared on the SiO_2_/Si substrate. At x = 1 mm (Figure 3d), the generated MoS_2_ domain appeared as small triangles and darts discretely. The triangular side of the largest domain reached up to 7 μm. At x = 1.5 mm (Figure 3e), with a larger domain size, regular triangles were formed. The side length of the largest triangular domain is about 15 μm. At x = 2.5 mm (Figure 3f), it is observed that part of MoS_2_ triangles are connected together to form some irregular film. The largest side of the triangular domain in this area is above 20 μm. Furthermore, large-scale MoS_2_ film has continuous coverage in the range of x = 3.5 to 13.5 mm (Figure 3g). As depicted in Figure 3h–k, contrary to the distribution in Figure 3c–f, the size of MoS_2_ samples decreases with the further increase of x. From y = 0 to 6.2 mm, a similar distribution of the MoS_2_ samples can be found along the x direction.

The differences of MoS_2_ domains in the direction vertical to the airflow (x-axis) are shown distinctly in the above OM images. Along the x-axis, the shapes of MoS_2_ were changed in the following order: small nucleated particles, small triangles, larger triangles, then large-sized film. Then, the film size gradually shrunk and became sparser. From Figure 3c–f, it can be inferred that that the vapor concentration of MoS_2_ increased continuously along the positive direction of the x-axis before MoS_2_ film formation. In Figure 3d, MoS_2_ domains would start growing from hexagonal nuclei with three Mo-zz and three S-zz sides. In this area, the Mo:S ratio condition was <1:2 so that small triangles and darts formed. From Figure 3f–h, it can be seen that a sufficient supply of MoS_2_ vapor results in large triangles and continuous film. In this area, the Mo:S ratio condition was ≥1:2 [27]. After the vapor concentration of MoS_2_ reached the maximum (i.e., filming phenomenon occurring), the MoS_2_ layer began to become discontinuous with the growth of the x-axis (Figure 3h–k), presenting a relatively sparse, discrete distribution of triangular MoS_2_ films. Meanwhile, there was a shrinkage in size and quantity with respect to these triangles. Based on the above analysis, we can deduce that there was an obviously a gradient distribution of MoS_2_ domain size in a cross-section vertical to the direction of airflow due to the difference of MoS_2_ vapor concentration.

To explore the size distribution rule of the MoS_2_ domain on the substrate, we chose five sections, as shown in Figure 3e–i. Each section had the same area (20,164 μm^2^) and labeled as Sections 1–5. The number of effective nucleation points (i.e., the nucleation points with MoS_2_ geometric area greater or equal to 0.5 μm^2^) and the nucleation density (i.e., the number of effective nucleation points per unit area) within the chosen section were statistically measured. According to statistical numbers in Table 1, the highest nucleation density is in Section 3, similar to the optical micrograph in Figure 3g. This area has the largest size of MoS_2_ film. Thus, the distribution rule of MoS_2_ domains on the substrate can be summarized as follows. For the same substrate along the direction vertical to the airflow, the nucleation density is related to the distance of the midcourt-line position of the substrate. The size of MoS_2_ thin film is larger as the distance is closer. Inversely, the larger the distance is, the smaller the size of the MoS_2_ thin film is and the fewer nucleation density of MoS_2_ is.

To explain the relationship between the nucleation density of MoS_2_ and the size distribution of MoS_2_ domains, we employed the laminar flow theory to analyze the airflow distribution. The largest speed of carrier airflow occurred in the center of the quartz tube, and the speed near the inwall of the quartz tube is close to zero [28,29]. Thus, along the direction vertical to the airflow, the speed of the carrier gas (Ar) is larger at the center surface of substrates than that their side. Since faster carrier gasses can transport more reactants in the same time, the area with a faster carrier gas can form more effective nucleation points, resulting in an increase of the crystal growth size [30]. As depicted in Figure 3g, the nucleation density also increased in the center region of substrates and MoS_2_ film formed. On the contrary, the area with a slower carrier gas can form less effective nucleation points, resulting in a decrease of nucleation density. As shown in Figure 3g, it is noted that a high nucleation density will increase growth points in the center area. An abundant supply of MoS_2_ vapor will make isolated MoS_2_ domains connect together. As shown in Figure 3e,i, when close to the side of the substrates, there is lower nucleation density, and enough MoS_2_ vapor enables the growth of larger sized triangles in this area. Furthermore, when at the side of the substrates, the lack of nuclei and vapor of MoS_2_ led to small triangles and discrete darts. Thus, domain morphology is highly dependent on nucleation density [31]. Thus, controlling the speed of carrier gas will be an effective approach for regulating nucleation density. Using this approach can also adjust the formation of MoS_2_ domains [19,22].

**Figure 4:**
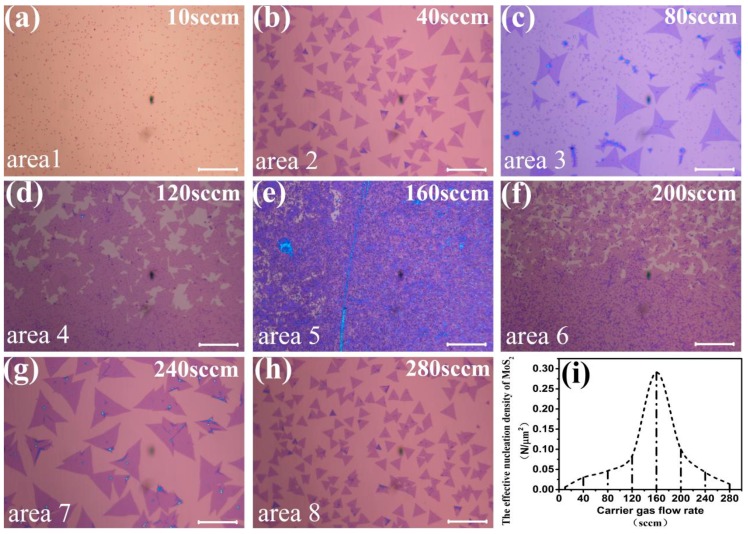
Effects of different carry gas flow rate on the effective nucleation density of MoS_2_. (**a**–**h**) MoS_2_ optical microscopy images of samples grown on the SiO_2_/Si substrates with different gas flow rates: 10, 40, 80, 120, 160, 200, 240, and 280 sccm. Scale bar: 20 μm. (**i**) The relationship diagram between the different carry gas flow rates (**a**–**h**) and the corresponding nucleation density of MoS_2_.

To further explore the influence of carrier gas flow rates on the nucleation density, we prepared MoS_2_ samples under the same conditions with different carrier gas flow rates, from 10, 40, 80, 120, 160, 200, to 240 sccm. Optical micrographs of MoS_2_ samples in the center point (x = 7.5 mm, y = 7.0 mm) of substrates with different carrier gas flow rates are shown in Figure 4a–h. The relationship between different carrier gas flow rates and their corresponding nucleation density is shown in Figure 4i. While the gas flow rate was 10 sccm, the nucleation density of MoS_2_ was 0.0061 N/μm^2^. Only MoS_2_ nuclei were found on substrates (Figure 4a). Although low gas flow rates lead to high concentrations of S vapor to fully sulfurize MoO_3_, it suffers from low transfer efficiency of the MoS_2_ vapor. As a result, few MoS_2_ nuclei can be deposited on substrates. By increasing gas flow rates in the range of 0–160 sccm, the concentration of S vapor decreased, but the concentration of MoS_2_ vapor was still enough. Therefore, the nucleation density of MoS_2_ increased. Thus, the growth of MoS_2_ was promoted in low flow rates. While the gas flow rate was 160 sccm, nucleation density reached the top value of 0.2912 N/μm^2^ and large-scale films of MoS_2_ were formed (Figure 4e). Even gas flow rates further increased from 160 to 280 sccm. The concentration of S vapor was not enough to maintain reactions of MoS_2_ synthesizing [32]. Therefore, the nucleation density of MoS_2_ decreased and the shape of MoS_2_ domains changed from disconnected film, to large triangles, then to small triangles (Figure 4f–h). Thus, the growth of MoS_2_ was suppressed in high flow rates. Finally, according to these experiment results, controlling the carrier gas flow rate can also control the shape and coverage of MoS_2_ domains.

## 4. Conclusions

In summary, we have shown a simple CVD method to synthesize 2D MoS_2_ crystals at atmospheric pressure. The results show that the size of MoS_2_ domains is 10 μm with fast growth. Raman, PL, and AFM measurements demonstrate that the inner region of triangular MoS_2_ domains were monolayer and the edge region were few-layer. It is observed that the shape of MoS_2_ domains vary from discrete darts, to triangles, to continuous film on the substrate. We also explore the distribution rule of synthesized MoS_2_ on SiO_2_/Si substrate. The nucleation density and the size and shape of MoS_2_ domains are related to the distance to the midcourt-line position of the substrate along the direction of the airflow. We employed the laminar flow theory to comprehend this distribution rule. It is noted that the changing the speed of the carrier gas at the substrate surface will control the nucleation density and adjust the formation of MoS_2_ domains. Furthermore, we explore the relationship between the carrier gas flow rate and the nucleation density. The results demonstrate that controlling the carrier gas flow rate will be an effective approach to control the size and coverage of MoS_2_ domains. It provides a valuable reference to understand the growth regulation of 2D MoS_2_.

## Figures and Tables

**Table 1:** A comparison of the number of effective nucleation points and the effective nucleation density of MoS_2_ in the same area of different regions.

Section	The Number of Effective Nucleation Points of MoS_2_ (N)	The Effective Nucleation Density of MoS_2_ (N/μm^2^)
Section 1 (Figure 3e)	207	0.0103
Section 2 (Figure 3f)	336	0.0167
Section 3 (Figure 3g)	784	0.0389
Section 4 (Figure 3h)	608	0.0302
Section 5 (Figure 3i)	72	0.0036
